# Crystal structure of (±)-(3a*R*,5*R*,8b*R*)-5-hydroper­oxy-2-phenyl-6-tosyl-4,5,6,8b-tetra­hydro­pyrrolo­[3,4-*e*]indole-1,3(2*H*,3a*H*)-dione

**DOI:** 10.1107/S1600536814019874

**Published:** 2014-09-13

**Authors:** Wayland E. Noland, Glen C. Gullickson, Rozalin R. Dickson, Leah L. Groess, Kenneth J. Tritch

**Affiliations:** a Department of Chemistry, University of Minnesota, Minneapolis, MN 55455-0431, USA

**Keywords:** crystal structure, hydro­peroxide, autoxidation, cyclo­addition, pyrrole, indole

## Abstract

The title compound, C_23_H_20_N_2_O_6_S, crystallizes as a racemate in the space group *P*-1, with an overall L- or J-shape to each mol­ecule. Centrosymmetric pairs of mol­ecules are tandem hydrogen bonded between the hydro­per­oxy H atom and carbonyl O atom. A different centrosymmetric pairing has stacked *S*-tolyl rings, and a third pairing is L,J-inter­locked by the short leg. Except for stacked tolyl pairs, neighboring π-systems are much closer to orthogonal than coaxial. The title compound is the first example of a hydro­peroxide obtained from the autoxidation of a Diels–Alder adduct of a 2-vinyl­pyrrole.

## Abstract 2

The title compound crystallized as racemic tandem OO—H⋯O=C hydrogen-bonded dimers stacked along [100] in the space group *P* 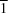. This is the first crystallographically characterized example of a hydro­peroxide obtained from the autoxidation of a Diels–Alder adduct of a 2-vinyl five-membered heterocycle.

## Chemical context   

Diels–Alder reactions of vinyl­pyrroles with male­imides have been studied as a method to form substituted indole compounds (Noland *et al.*, 2009 ▶; Xiao & Ketcha, 1995 ▶). Related reactions have been done with other heterocycles (Abarca *et al.*, 1985 ▶; Jones *et al.*, 1984 ▶; Noland *et al.*, 1983 ▶). Diels–Alder reactions between vinyl­heterocycles and dienophiles are useful for forming fused ring systems that may have biological activity or versatility in natural product synthesis (Booth *et al.*, 2005 ▶; Kanai *et al.* 2005 ▶; Nagai *et al.* 1993 ▶; Noland & Pardi, 2005 ▶).
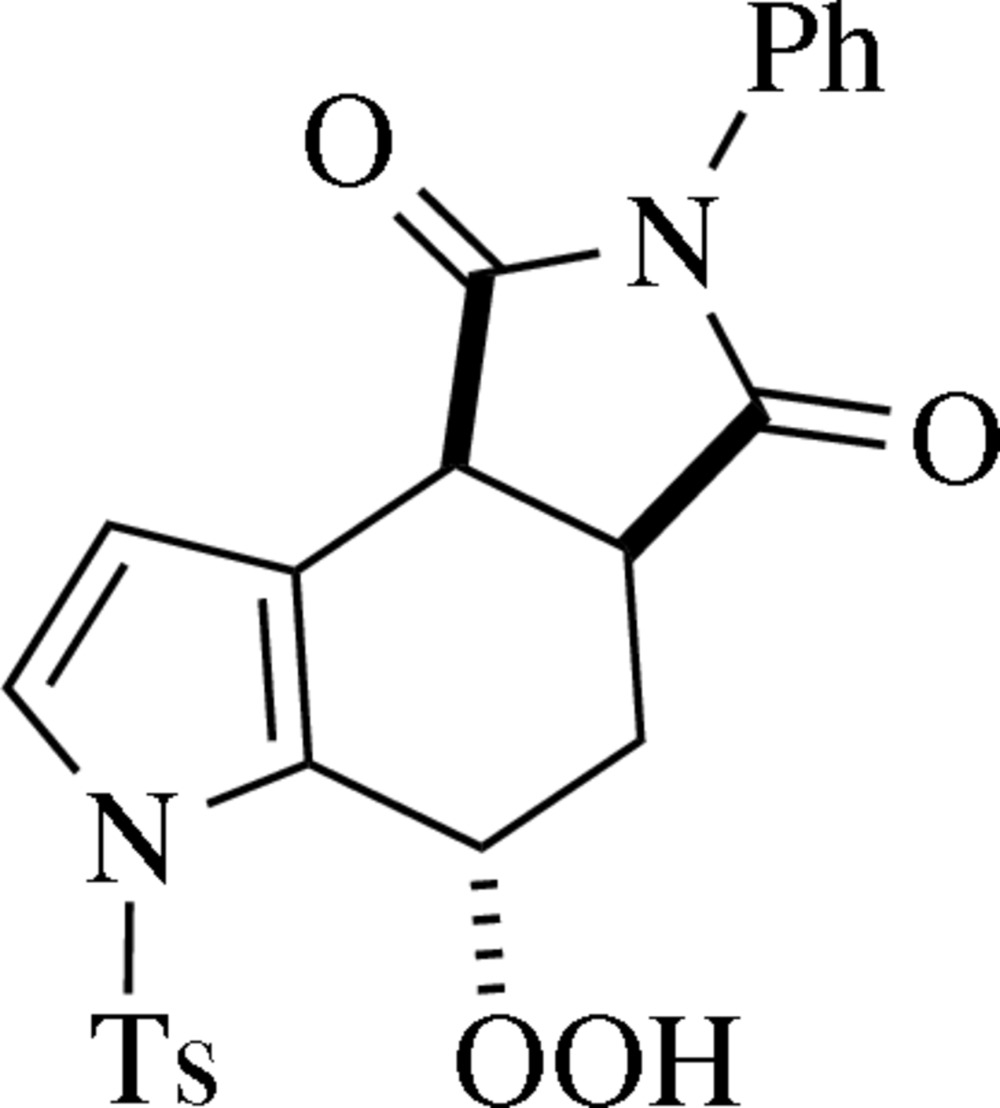


**Figure 1:**
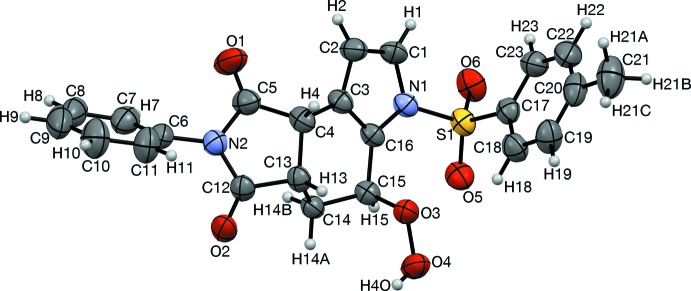
The mol­ecular structure of (I) with displacement ellipsoids drawn at the 50% probability level.

The hydro­peroxide title compound (I) (Fig. 1 ▶) was isolated after performing a Diels–Alder reaction between *N*-tosyl-2-vinyl­pyrrole (Settambolo *et al.*, 1997 ▶) and *N*-phenyl­male­imide (commercially available). An analogous compound was proposed, though not isolated, as an inter­mediate for an alcohol product obtained from a similar reaction (Eitel & Pindur, 1988 ▶). No analogous hydro­peroxides have been claimed from reactions of 2-vinyl -pyrroles, -indoles, -thio­phenes, or -benzo­thio­phenes. There are a few examples of hydro­peroxides isolated from Diels–Alder reactions between 2-vinyl­furan or 2-vinyl­benzo­furan and dienophiles, but the products were not crystallographically categorized (Brewer & Elix, 1975 ▶; Kotsuki *et al.*, 1981 ▶; Skoric *et al.*, 2001 ▶).

## Structural commentary   

**Figure 2:**
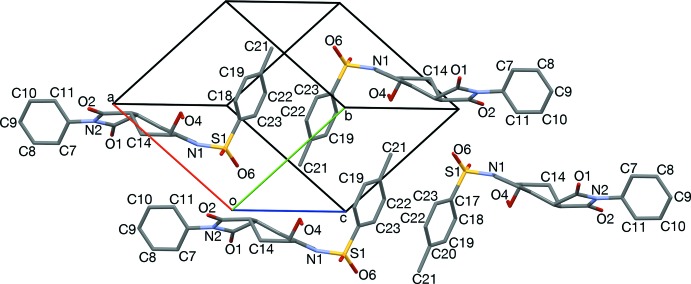
Two pairs of stacked tolyl groups, viewed along [1
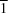
2]. The central two mol­ecules form an inter­locked pair. Twisting of the *N*-Phenyl group, and out-of-plane position of C14, are also depicted.

The *N*-phenyl, *S*-phenyl, and pyrrolo rings are individually planar within 0.009, 0.011, and 0.010 Å, respectively. The *N*-phenyl ring (C7–C11) is twisted 58.3 (2)° out of plane from the imido moiety (C5, N2, C12, Figs. 2 ▶ and 4 ▶). The cyclo­hexene ring has a half-chair conformation with C14 out of plane in the direction *anti*- to the *S*-tolyl group (Figs. 2 ▶ and 4 ▶), which is bent 85.4 (2)° out of plane with the pyrrolo ring, giving the mol­ecule an overall L- or J- shape.

## Supra­molecular features   

**Figure 3:**
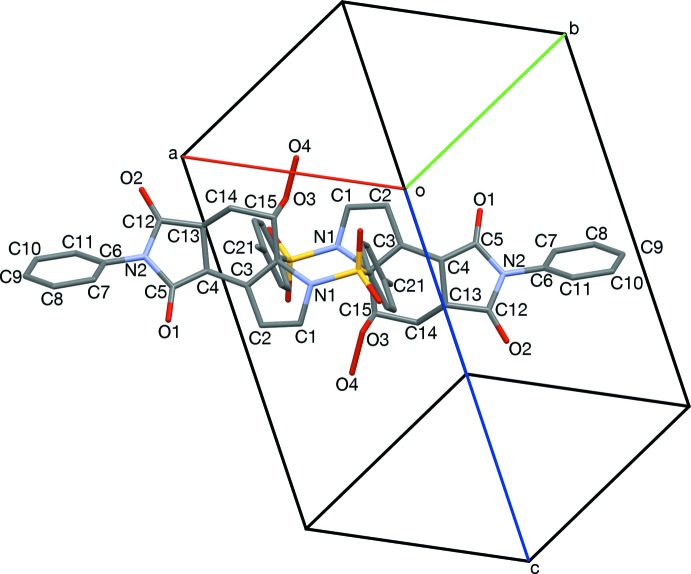
The central inter­locked pair from Fig. 2 ▶, viewed along [221]. The C20,C21-axis is aligned with the face of the cyclo­hexene ring of its inter­locked partner.

**Figure 4:**
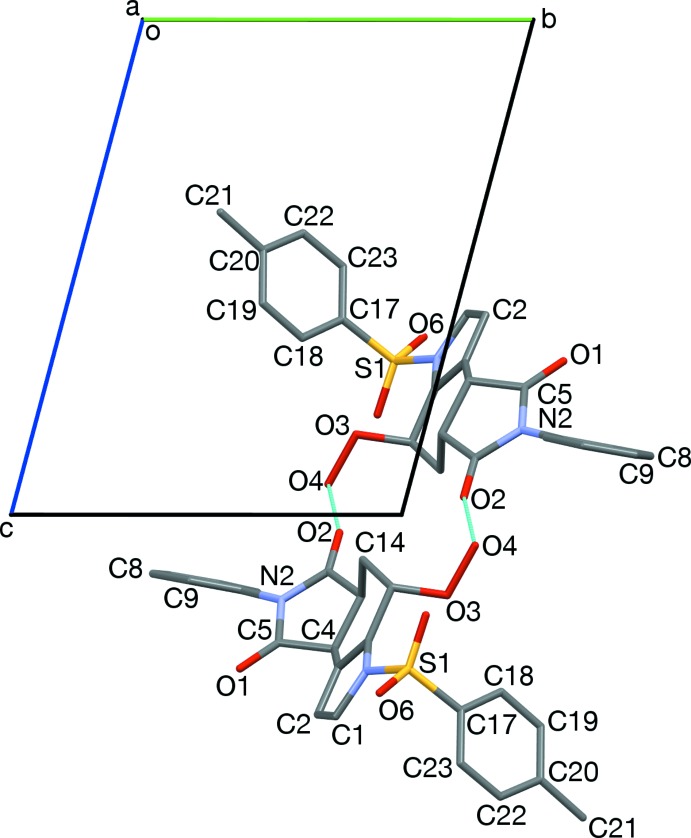
Hydrogen-bonded dimers, viewed along [100]. Hydrogen bonds (O2, O4) are shown in turquoise. Also apparent are the twisting of *N*-phenyl rings (C5, N2, C8, C9), and the half-chair conformation of the cyclo­hexene ring (C4, C14, lower mol­ecule).

**Figure 6:**
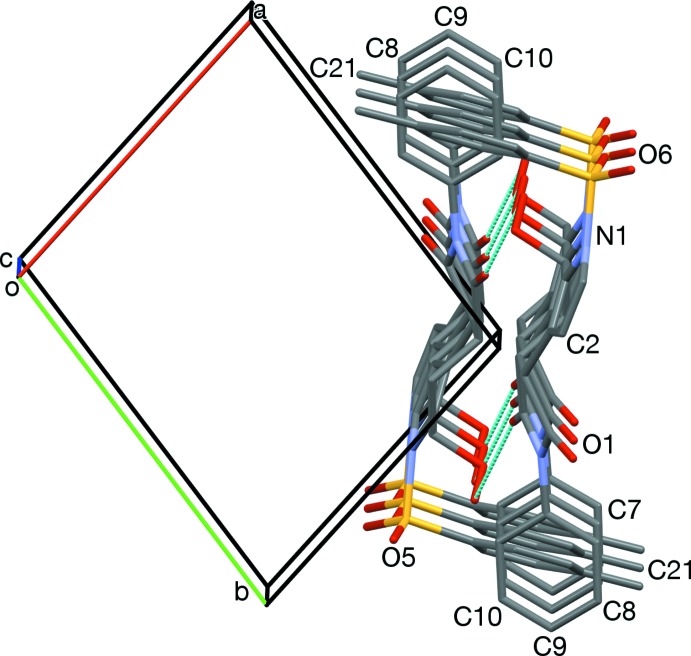
Edge-to-face stacks of *S*-tolyl and *N*-phenyl rings, and hydrogen-bonded (turquoise) dimerization, viewed slightly off [001].

**Figure 7:**
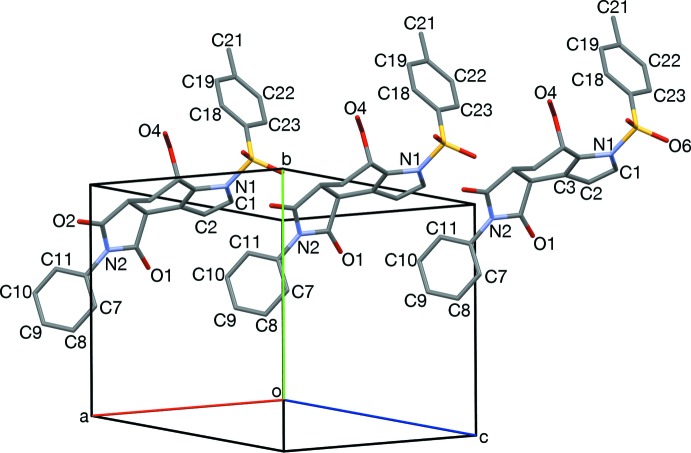
The *endo* face of pyrrole rings (N1, C1, C2) neighboring the edge of an *N*-phenyl ring (C10, C11) of an adjacent mol­ecule of the same enanti­omer, viewed along [414].

Inter­locking pairs are aligned such that the axis of the *S*-tolyl group (C21) points toward the face of the cyclo­hexene ring (C3, C14, C15, Fig. 3 ▶). Hydrogen-bonded dimers form between H4*O* and O2 (Table 1 ▶, Figs. 4 ▶ and 6 ▶). Hydrogen bonding acts approximately along [4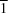 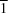] and twists the hydro­per­oxy group to have a torsion angle of 95.0°. In different pairings than those that inter­lock, *S*-tolyl groups stack rotated 180° about an oblique axis, [0.803, −0.544, 0.244] (Figs. 2 ▶ and 5 ▶). Each pair of *S*-tolyl pairs is sheared by approximately 3.7 Å from its neighbor. Similarly oriented *N*-phenyl rings are separated from each other by *S*-tolyl groups (C21, Fig. 6 ▶), with an angle of 69.26° between the *S*-tolyl and *N*-phenyl planes. Pyrrolo groups (N1, C1, C2) each have their *endo* face toward an edge of an *N*-phenyl group (C10, C11, Fig. 7 ▶), with an angle of 69.4 (2)° between the pyrrolo and *N*-phenyl planes.

## Database survey   

**Figure 8:**
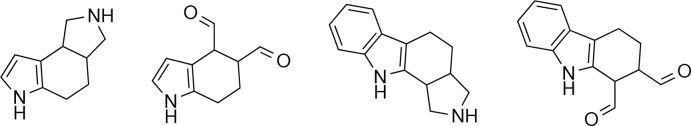
Substructures used for the database survey.

The structures shown in Fig. 8 ▶ represent the cores of most compounds that could be obtained by Diels–Alder reactions of the type that gave title compound (I), using nitro­gen heterocyclic dienes and dienophiles. Searching these substructures found six entries in the current database (Cambridge Structural Database, Version 5.35, November 2013; Allen 2002 ▶). Only two of these were synthesized by cyclo­additions of this type [a combretastatin derivative (Ty *et al.*, 2010 ▶); carbazomycin B (Beccali *et al.*, 1996 ▶)]. Upon expanding the search to include any combination of heteroatoms at nitro­gen and oxygen sites, seven additional entries were found, all within or closely related to the caesalmin family of furan­oditerpenoid anti­virals (*i.e.*, Rodrigues *et al.*, 2004 ▶; Jiang *et al.*, 2002 ▶). Of 13 total entries, none was found containing sulfur atoms. Ten exhibit inter­molecular hydrogen bonding, but only two of them are tandem-bonded dimers [pyrimidinone carbonyl to carb­oxy­lic acid (Obushak *et al.*, 2011 ▶); succinimide dimerization between N-H and a carbonyl oxygen (Beccali *et al.*, 1996 ▶)]. None of these structures resembles that of compound (I).

## Synthesis and crystallization   

*N*-Tosyl-2-vinyl­pyrrole (458 mg) and *N*-phenyl­male­imide (272 mg) were dissolved in chloro­form (1.5 ml) and stirred for 72 h at room temperature in a vessel open to air. Column chromatography on silica gel (1:1 hexa­ne:ethyl acetate, *R_f_* = 0.30), followed by recrystallization from di­chloro­methane–petroleum ether (b.p. 311–333 K) gave compound (I) as colourless plates (17 mg, 2.4%, m.p. 425–426 K). ^1^H NMR (500 MHz, (CD_3_)_2_SO) δ 11.96 (*s*, 1H, H4*O*), 7.974 (*d*, *J* = 3.4 Hz, 2H, H18, H23), 7.483 (*t*, *J* = 8.0 Hz, 2H, H8, H10), 7.427 (*m*, 3H, H9, H19, H22), 7.283 (*dd*, *J* = 8.8, 1.0 Hz, 2H, H7, H11), 6.516 (*d*, *J* = 3.4 Hz, 1H, H2), 5.485 (*dd*, *J* = 2.9, 2.7 Hz, 1H, H15), 4.241 (*d*, *J* = 8.3 Hz, 1H, H4), 3.375 (*ddd*, *J* = 13.7, 8.3, 5.8 Hz, 1H, H13), 2.824 (*ddd*, *J* = 14.1, 5.8, 2.9 Hz, 1H, H14*A*), 2.384 (*s*, 3H, H21), 1.886 (*ddd*, *J* = 14.1, 13.7, 2.7 Hz, 1H, H14*B*); ^13^C NMR (126 MHz, (CD_3_)_2_SO) δ 178.09 (C12), 174.85 (C5), 145.49 (C20), 135.06 (C17), 132.36 (C6), 129.94 (C19, C22), 128.87 (C8, C10), 128.43 (C9), 127.57 (C18, C23), 127.26 (C7, C11), 125.32 (C16), 124.14 (C1), 121.63 (C3), 112.27 (C2), 70.77 (C15), 38.79 (C4), 35.32 (C13), 27.85 (C14), 21.10 (C21); IR (NaCl, cm^−1^) 3361 (O–H), 2917 (C–H), 1713 (C=O), 1595 (C=C), 1370 (S=O), 1177 (C–O), 808, 751, 672; MS (ESI, PEG, *m/z*) [M+Na]^+^ calculated for C_23_H_20_N_2_O_6_S 475.0934, found 475.0921.

**Safety Note:** Hydro­peroxides, particularly those bearing an α-proton (*e.g.*, H15, Fig. 1 ▶), can be shock- or heat-sensitive and detonate violently (Francisco, 1993 ▶). Although sensitivity tests on our batch of title compound (I) were negative, appropriate precautions should be taken when reproducing or extending our work with (I) or similar compounds. Metal tools, glass or metal storage vessels, high oxygen- or nitro­gen-to-carbon ratios, and large scales should all be avoided. The bulk of product should be kept in solution, with small aliquots being allowed to dry only as necessary.

## Refinement   

A direct-methods solution was calculated which provided most non-hydrogen atoms from the E-map. Full-matrix least-squares/difference Fourier cycles were performed, which located the remaining non-hydrogen atoms. All non-hydrogen atoms were refined with anisotropic displacement parameters. All hydrogen atoms except for H4*O* were placed in ideal positions and refined as constrained atoms with *U*_iso_(H_*n*_) = 1.2*U*_eq_(C_*n*_), except for the methyl group, where *U*_iso_(H21_i_) = 1.5*U*_eq_(C21). The bond lengths (Å) specified for C–H hydrogens were 0.93 (ar­yl), 0.96 (meth­yl), 0.97 (methyl­ene), and 0.98 (methine). H4*O* was found from the difference Fourier map and refined with *U*_iso_(H4*O*) = 1.2*U*_eq_(O4) and an O—H bond length of 0.82 Å. The final full-matrix least-squares refinement converged to *R*1 = 0.0364 and *wR*2 = 0.1161 (*F* ^2^, all data). Crystal data, data collection and structure refinement details are summarized in Table 2 ▶.

## Supplementary Material

Crystal structure: contains datablock(s) global, I. DOI: 10.1107/S1600536814019874/hb7256sup1.cif

Structure factors: contains datablock(s) I. DOI: 10.1107/S1600536814019874/hb7256Isup2.hkl

Supporting information file. DOI: 10.1107/S1600536814019874/hb7256Isup3.cml

CCDC reference: 1022638

Additional supporting information:  crystallographic information; 3D view; checkCIF report

## Figures and Tables

**Figure 5:**
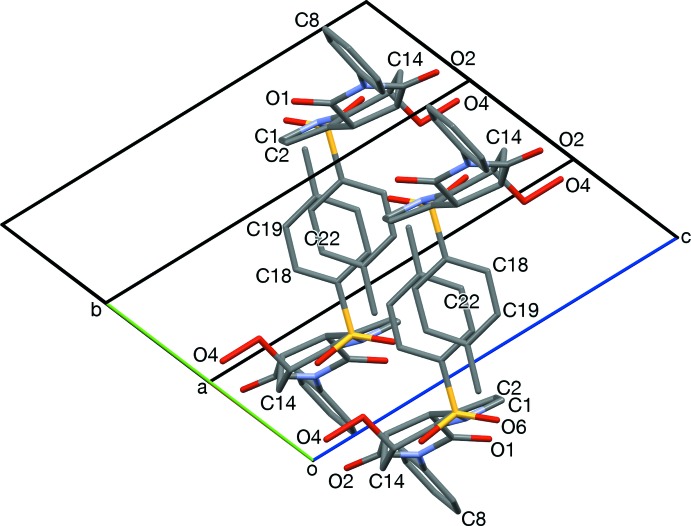
The two tolyl-stacked pairs from Fig. 2 ▶, viewed along [2
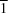
0]. Neighboring pairs are sheared roughly 1.5 phenyl ring diameters.

**Table 1:** Hydrogen-bond geometry (Å, °)

*D*—H⋯*A*	*D*—H	H⋯*A*	*D*⋯*A*	*D*—H⋯*A*
O4—H4*O*⋯O2^i^	0.82	2.00	2.7929 (19)	163

Symmetry code: (i) 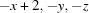.

**Table 2:** Experimental details

Crystal data	
Chemical formula	C_23_H_20_N_2_O_6_S
*M*_r_	452.48
Crystal system, space group	Triclinic, *P* 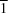
Temperature (K)	293
*a*, *b*, *c* (Å)	8.6563 (13), 9.8819 (15), 13.533 (2)
α, β, γ (°)	102.068 (3), 107.786 (2), 96.364 (2)
*V* (Å^3^)	1058.8 (3)
*Z*	2
Radiation type	Mo *K*α
μ (mm^−1^)	0.20
Crystal size (mm)	0.60 × 0.50 × 0.20

Data collection	
Diffractometer	Bruker SMART Platform CCD
Absorption correction	Multi-scan (*SADABS*; Blessing, 1995 ▶)
*T*_min_, *T*_max_	0.891, 0.962
No. of measured, independent and observed [*I* > 2σ(*I*)] reflections	10544, 3751, 3194
*R*_int_	0.021
(sin θ/λ)_max_ (Å^−1^)	0.596

Refinement	
*R*[*F* ^2^ > 2σ(*F* ^2^)], *wR*(*F* ^2^), *S*	0.036, 0.116, 1.06
No. of reflections	3751
No. of parameters	290
H-atom treatment	H-atom parameters constrained
Δρ_max_, Δρ_min_ (e Å^−3^)	0.27, −0.28

Computer programs: *SMART* (Bruker, 2001 ▶), *SAINT* (Bruker, 2003 ▶), *SHELXS97* and *SHELXL97* (Sheldrick, 2008 ▶), *Mercury* (Macrae *et al.*, 2008 ▶), *enCIFer* (Allen *et al.*, 2004 ▶), and *publCIF* (Westrip, 2010 ▶).

## supplementary crystallographic information

### Crystal data

**Table:**

C_23_H_20_N_2_O_6_S	*F*(000) = 472
*M**_r_* = 452.48	*D*_x_ = 1.419 Mg m^−^^3^
Triclinic, *P*1	Melting point: 425 K
*a* = 8.6563 (13) Å	Mo *K*α radiation, λ = 0.71073 Å
*b* = 9.8819 (15) Å	Cell parameters from 2698 reflections
*c* = 13.533 (2) Å	θ = 2.5–25.1°
α = 102.068 (3)°	µ = 0.20 mm^−^^1^
β = 107.786 (2)°	*T* = 293 K
γ = 96.364 (2)°	Block, colourless
*V* = 1058.8 (3) Å^3^	0.60 × 0.50 × 0.20 mm
*Z* = 2	

### Data collection

**Table:**

Bruker SMART Platform CCD diffractometer	3751 independent reflections
Radiation source: normal-focus sealed tube	3194 reflections with *I* > 2σ(*I*)
Graphite monochromator	*R*_int_ = 0.021
area detector, ω scans per phi	θ_max_ = 25.1°, θ_min_ = 1.6°
Absorption correction: multi-scan (*SADABS*; Blessing, 1995)	*h* = −10→10
*T*_min_ = 0.891, *T*_max_ = 0.962	*k* = −11→11
10544 measured reflections	*l* = −16→16

### Refinement

**Table:**

Refinement on *F*^2^	Primary atom site location: structure-invariant direct methods
Least-squares matrix: full	Secondary atom site location: difference Fourier map
*R*[*F*^2^ > 2σ(*F*^2^)] = 0.036	Hydrogen site location: inferred from neighbouring sites
*wR*(*F*^2^) = 0.116	H-atom parameters constrained
*S* = 1.06	*w* = 1/[σ^2^(*F*_o_^2^) + (0.0678*P*)^2^ + 0.2551*P*] where *P* = (*F*_o_^2^ + 2*F*_c_^2^)/3
3751 reflections	(Δ/σ)_max_ < 0.001
290 parameters	Δρ_max_ = 0.27 e Å^−^^3^
0 restraints	Δρ_min_ = −0.28 e Å^−^^3^

### Special details

**Table:**

Experimental. The space group P-1 was determined based on systematic absences and intensity statistics.
Geometry. All e.s.d.'s (except the e.s.d. in the dihedral angle between two l.s. planes) are estimated using the full covariance matrix. The cell e.s.d.'s are taken into account individually in the estimation of e.s.d.'s in distances, angles and torsion angles; correlations between e.s.d.'s in cell parameters are only used when they are defined by crystal symmetry. An approximate (isotropic) treatment of cell e.s.d.'s is used for estimating e.s.d.'s involving l.s. planes.
Refinement. Refinement of *F*^2^ against ALL reflections. The weighted *R*-factor *wR* and goodness of fit *S* are based on *F*^2^, conventional *R*-factors *R* are based on *F*, with *F* set to zero for negative *F*^2^. The threshold expression of *F*^2^ > σ(*F*^2^) is used only for calculating *R*-factors(gt) *etc*. and is not relevant to the choice of reflections for refinement. *R*-factors based on *F*^2^ are statistically about twice as large as those based on *F*, and *R*- factors based on ALL data will be even larger.

### Fractional atomic coordinates and isotropic or equivalent isotropic displacement parameters (Å^2^)

**Table:**

	*x*	*y*	*z*	*U*_iso_*/*U*_eq_	
S1	0.46933 (5)	0.12220 (5)	0.31067 (4)	0.04468 (16)	
O1	1.0118 (2)	−0.3093 (2)	0.31048 (15)	0.0865 (6)	
O2	1.09502 (19)	−0.14706 (14)	0.03647 (12)	0.0582 (4)	
O3	0.74791 (17)	0.17137 (13)	0.16243 (10)	0.0499 (3)	
O4	0.6719 (2)	0.20863 (16)	0.06212 (12)	0.0649 (4)	
H4O	0.7339	0.2033	0.0268	0.078*	
O5	0.40641 (16)	0.13010 (15)	0.20262 (10)	0.0566 (4)	
O6	0.36195 (16)	0.06483 (15)	0.35954 (12)	0.0601 (4)	
N1	0.60788 (18)	0.01608 (15)	0.31539 (12)	0.0418 (3)	
N2	1.08339 (19)	−0.24962 (16)	0.17207 (12)	0.0474 (4)	
C1	0.6732 (2)	−0.0313 (2)	0.40679 (15)	0.0480 (4)	
H1	0.6284	−0.0301	0.4612	0.058*	
C2	0.8110 (2)	−0.0788 (2)	0.40345 (15)	0.0502 (5)	
H2	0.8770	−0.1182	0.4539	0.060*	
C3	0.8381 (2)	−0.05797 (19)	0.30826 (13)	0.0420 (4)	
C4	0.9808 (2)	−0.0811 (2)	0.27117 (14)	0.0457 (4)	
H4	1.0786	−0.0165	0.3247	0.055*	
C5	1.0216 (2)	−0.2278 (2)	0.25780 (16)	0.0539 (5)	
C6	1.1781 (2)	−0.35732 (19)	0.15237 (15)	0.0486 (4)	
C7	1.1125 (3)	−0.4973 (2)	0.13290 (17)	0.0610 (5)	
H7	1.0042	−0.5252	0.1291	0.073*	
C8	1.2118 (4)	−0.5966 (2)	0.11898 (18)	0.0739 (7)	
H8	1.1700	−0.6916	0.1076	0.089*	
C9	1.3694 (4)	−0.5565 (3)	0.1218 (2)	0.0801 (8)	
H9	1.4336	−0.6239	0.1112	0.096*	
C10	1.4322 (4)	−0.4178 (3)	0.1400 (2)	0.0857 (8)	
H10	1.5393	−0.3904	0.1413	0.103*	
C11	1.3370 (3)	−0.3167 (2)	0.1568 (2)	0.0669 (6)	
H11	1.3811	−0.2217	0.1710	0.080*	
C12	1.0551 (2)	−0.14946 (18)	0.11427 (15)	0.0443 (4)	
C13	0.9635 (2)	−0.04746 (18)	0.16302 (14)	0.0429 (4)	
H13	1.0188	0.0496	0.1752	0.051*	
C14	0.7826 (2)	−0.06872 (18)	0.09075 (14)	0.0427 (4)	
H14A	0.7783	−0.0455	0.0239	0.051*	
H14B	0.7310	−0.1668	0.0739	0.051*	
C15	0.6877 (2)	0.02411 (18)	0.14624 (13)	0.0415 (4)	
H15	0.5697	0.0001	0.1039	0.050*	
C16	0.7143 (2)	0.00040 (17)	0.25498 (13)	0.0378 (4)	
C17	0.5829 (2)	0.28523 (19)	0.39512 (14)	0.0435 (4)	
C18	0.6708 (3)	0.3751 (2)	0.35718 (17)	0.0550 (5)	
H18	0.6656	0.3521	0.2858	0.066*	
C19	0.7667 (3)	0.5001 (2)	0.42738 (19)	0.0618 (6)	
H19	0.8253	0.5616	0.4024	0.074*	
C20	0.7770 (3)	0.5351 (2)	0.53416 (18)	0.0592 (5)	
C21	0.8857 (3)	0.6697 (3)	0.6109 (2)	0.0849 (8)	
H21A	0.9501	0.6498	0.6760	0.127*	
H21B	0.8180	0.7359	0.6270	0.127*	
H21C	0.9582	0.7091	0.5785	0.127*	
C22	0.6853 (3)	0.4442 (2)	0.56897 (17)	0.0623 (6)	
H22	0.6886	0.4682	0.6399	0.075*	
C23	0.5893 (3)	0.3192 (2)	0.50137 (16)	0.0561 (5)	
H23	0.5297	0.2585	0.5264	0.067*	

### Atomic displacement parameters (Å^2^)

**Table:**

	*U*^11^	*U*^22^	*U*^33^	*U*^12^	*U*^13^	*U*^23^
S1	0.0331 (2)	0.0524 (3)	0.0469 (3)	0.00619 (19)	0.01535 (19)	0.0075 (2)
O1	0.1099 (14)	0.1116 (14)	0.0929 (12)	0.0657 (12)	0.0665 (11)	0.0702 (12)
O2	0.0736 (9)	0.0576 (8)	0.0673 (9)	0.0237 (7)	0.0473 (8)	0.0254 (7)
O3	0.0635 (8)	0.0467 (7)	0.0459 (7)	0.0136 (6)	0.0247 (6)	0.0140 (6)
O4	0.0787 (10)	0.0795 (10)	0.0667 (9)	0.0402 (8)	0.0424 (8)	0.0423 (8)
O5	0.0447 (7)	0.0696 (9)	0.0483 (8)	0.0146 (6)	0.0084 (6)	0.0091 (6)
O6	0.0425 (7)	0.0688 (9)	0.0723 (9)	0.0034 (6)	0.0314 (7)	0.0108 (7)
N1	0.0391 (8)	0.0465 (8)	0.0433 (8)	0.0059 (6)	0.0196 (6)	0.0115 (6)
N2	0.0507 (9)	0.0484 (9)	0.0504 (9)	0.0150 (7)	0.0236 (7)	0.0157 (7)
C1	0.0546 (11)	0.0550 (11)	0.0443 (10)	0.0098 (9)	0.0266 (9)	0.0189 (8)
C2	0.0535 (11)	0.0612 (12)	0.0418 (10)	0.0152 (9)	0.0183 (9)	0.0200 (9)
C3	0.0367 (9)	0.0502 (10)	0.0377 (9)	0.0062 (7)	0.0133 (7)	0.0079 (7)
C4	0.0361 (9)	0.0583 (11)	0.0408 (9)	0.0088 (8)	0.0130 (7)	0.0088 (8)
C5	0.0495 (11)	0.0702 (13)	0.0537 (11)	0.0214 (10)	0.0238 (9)	0.0255 (10)
C6	0.0549 (11)	0.0473 (10)	0.0450 (10)	0.0172 (8)	0.0162 (8)	0.0120 (8)
C7	0.0657 (13)	0.0542 (12)	0.0545 (12)	0.0097 (10)	0.0096 (10)	0.0126 (9)
C8	0.106 (2)	0.0464 (12)	0.0550 (13)	0.0230 (13)	0.0084 (13)	0.0055 (10)
C9	0.093 (2)	0.0737 (17)	0.0715 (16)	0.0450 (15)	0.0204 (14)	0.0114 (13)
C10	0.0717 (16)	0.0843 (19)	0.108 (2)	0.0351 (14)	0.0369 (15)	0.0195 (16)
C11	0.0604 (13)	0.0548 (12)	0.0870 (16)	0.0165 (10)	0.0291 (12)	0.0117 (11)
C12	0.0451 (10)	0.0424 (9)	0.0510 (10)	0.0071 (8)	0.0240 (8)	0.0127 (8)
C13	0.0429 (9)	0.0414 (9)	0.0484 (10)	0.0062 (7)	0.0227 (8)	0.0101 (8)
C14	0.0502 (10)	0.0423 (9)	0.0373 (9)	0.0078 (8)	0.0185 (8)	0.0086 (7)
C15	0.0393 (9)	0.0458 (9)	0.0382 (9)	0.0061 (7)	0.0136 (7)	0.0087 (7)
C16	0.0339 (8)	0.0419 (9)	0.0367 (8)	0.0019 (7)	0.0148 (7)	0.0065 (7)
C17	0.0371 (9)	0.0475 (10)	0.0458 (10)	0.0115 (8)	0.0136 (8)	0.0105 (8)
C18	0.0590 (12)	0.0555 (11)	0.0552 (11)	0.0105 (9)	0.0291 (10)	0.0096 (9)
C19	0.0603 (13)	0.0477 (11)	0.0815 (15)	0.0054 (9)	0.0356 (11)	0.0105 (10)
C20	0.0478 (11)	0.0485 (11)	0.0684 (14)	0.0165 (9)	0.0076 (10)	0.0023 (10)
C21	0.0708 (16)	0.0581 (14)	0.100 (2)	0.0107 (12)	0.0115 (14)	−0.0084 (13)
C22	0.0750 (15)	0.0585 (12)	0.0453 (11)	0.0179 (11)	0.0106 (10)	0.0079 (9)
C23	0.0663 (13)	0.0558 (12)	0.0466 (11)	0.0108 (10)	0.0192 (10)	0.0142 (9)

### Geometric parameters (Å, º)

**Table:**

S1—O5	1.4182 (14)	C8—H8	0.9300
S1—O6	1.4278 (14)	C9—C10	1.359 (4)
S1—N1	1.6740 (15)	C9—H9	0.9300
S1—C17	1.7494 (19)	C10—C11	1.389 (3)
O1—C5	1.194 (2)	C10—H10	0.9300
O2—C12	1.208 (2)	C11—H11	0.9300
O3—C15	1.437 (2)	C12—C13	1.516 (2)
O3—O4	1.4590 (18)	C13—C14	1.536 (3)
O4—H4O	0.8200	C13—H13	0.9800
N1—C1	1.393 (2)	C14—C15	1.528 (2)
N1—C16	1.407 (2)	C14—H14A	0.9700
N2—C12	1.383 (2)	C14—H14B	0.9700
N2—C5	1.407 (2)	C15—C16	1.491 (2)
N2—C6	1.443 (2)	C15—H15	0.9800
C1—C2	1.340 (3)	C17—C18	1.382 (3)
C1—H1	0.9300	C17—C23	1.389 (3)
C2—C3	1.429 (2)	C18—C19	1.384 (3)
C2—H2	0.9300	C18—H18	0.9300
C3—C16	1.356 (2)	C19—C20	1.386 (3)
C3—C4	1.492 (2)	C19—H19	0.9300
C4—C5	1.517 (3)	C20—C22	1.381 (3)
C4—C13	1.535 (2)	C20—C21	1.510 (3)
C4—H4	0.9800	C21—H21A	0.9600
C6—C11	1.369 (3)	C21—H21B	0.9600
C6—C7	1.373 (3)	C21—H21C	0.9600
C7—C8	1.396 (3)	C22—C23	1.374 (3)
C7—H7	0.9300	C22—H22	0.9300
C8—C9	1.364 (4)	C23—H23	0.9300
			
O5—S1—O6	120.26 (9)	C10—C11—H11	120.1
O5—S1—N1	106.58 (8)	O2—C12—N2	124.70 (16)
O6—S1—N1	104.10 (8)	O2—C12—C13	126.41 (16)
O5—S1—C17	110.99 (9)	N2—C12—C13	108.87 (15)
O6—S1—C17	108.79 (9)	C12—C13—C4	103.09 (14)
N1—S1—C17	104.77 (8)	C12—C13—C14	111.55 (14)
C15—O3—O4	107.67 (12)	C4—C13—C14	112.69 (14)
O3—O4—H4O	109.5	C12—C13—H13	109.8
C1—N1—C16	108.04 (14)	C4—C13—H13	109.8
C1—N1—S1	120.64 (12)	C14—C13—H13	109.8
C16—N1—S1	127.91 (12)	C15—C14—C13	110.81 (14)
C12—N2—C5	112.50 (15)	C15—C14—H14A	109.5
C12—N2—C6	123.69 (15)	C13—C14—H14A	109.5
C5—N2—C6	123.61 (15)	C15—C14—H14B	109.5
C2—C1—N1	108.87 (15)	C13—C14—H14B	109.5
C2—C1—H1	125.6	H14A—C14—H14B	108.1
N1—C1—H1	125.6	O3—C15—C16	106.51 (13)
C1—C2—C3	107.45 (17)	O3—C15—C14	111.80 (14)
C1—C2—H2	126.3	C16—C15—C14	108.60 (14)
C3—C2—H2	126.3	O3—C15—H15	110.0
C16—C3—C2	108.74 (16)	C16—C15—H15	110.0
C16—C3—C4	121.81 (16)	C14—C15—H15	110.0
C2—C3—C4	129.26 (17)	C3—C16—N1	106.87 (15)
C3—C4—C5	117.02 (16)	C3—C16—C15	125.22 (15)
C3—C4—C13	113.64 (15)	N1—C16—C15	127.44 (15)
C5—C4—C13	104.65 (14)	C18—C17—C23	120.96 (18)
C3—C4—H4	107.0	C18—C17—S1	119.98 (14)
C5—C4—H4	107.0	C23—C17—S1	118.99 (15)
C13—C4—H4	107.0	C17—C18—C19	118.74 (19)
O1—C5—N2	124.96 (19)	C17—C18—H18	120.6
O1—C5—C4	128.61 (19)	C19—C18—H18	120.6
N2—C5—C4	106.36 (16)	C18—C19—C20	121.3 (2)
C11—C6—C7	120.53 (19)	C18—C19—H19	119.3
C11—C6—N2	118.49 (18)	C20—C19—H19	119.3
C7—C6—N2	120.95 (18)	C22—C20—C19	118.47 (19)
C6—C7—C8	118.6 (2)	C22—C20—C21	120.6 (2)
C6—C7—H7	120.7	C19—C20—C21	121.0 (2)
C8—C7—H7	120.7	C20—C21—H21A	109.5
C9—C8—C7	120.9 (2)	C20—C21—H21B	109.5
C9—C8—H8	119.5	H21A—C21—H21B	109.5
C7—C8—H8	119.5	C20—C21—H21C	109.5
C10—C9—C8	119.9 (2)	H21A—C21—H21C	109.5
C10—C9—H9	120.1	H21B—C21—H21C	109.5
C8—C9—H9	120.1	C23—C22—C20	121.6 (2)
C9—C10—C11	120.2 (3)	C23—C22—H22	119.2
C9—C10—H10	119.9	C20—C22—H22	119.2
C11—C10—H10	119.9	C22—C23—C17	118.9 (2)
C6—C11—C10	119.8 (2)	C22—C23—H23	120.5
C6—C11—H11	120.1	C17—C23—H23	120.5
			
O5—S1—N1—C1	−169.75 (14)	O2—C12—C13—C14	70.7 (2)
O6—S1—N1—C1	−41.65 (15)	N2—C12—C13—C14	−107.92 (17)
C17—S1—N1—C1	72.53 (15)	C3—C4—C13—C12	−148.89 (15)
O5—S1—N1—C16	33.70 (17)	C5—C4—C13—C12	−20.06 (18)
O6—S1—N1—C16	161.79 (14)	C3—C4—C13—C14	−28.5 (2)
C17—S1—N1—C16	−84.02 (16)	C5—C4—C13—C14	100.34 (17)
C16—N1—C1—C2	−1.8 (2)	C12—C13—C14—C15	174.11 (14)
S1—N1—C1—C2	−162.56 (14)	C4—C13—C14—C15	58.70 (19)
N1—C1—C2—C3	1.6 (2)	O4—O3—C15—C16	−159.65 (12)
C1—C2—C3—C16	−0.8 (2)	O4—O3—C15—C14	81.87 (16)
C1—C2—C3—C4	174.24 (19)	H4O—O4—O3—C15	−95.0
C16—C3—C4—C5	−128.24 (19)	C13—C14—C15—O3	65.49 (18)
C2—C3—C4—C5	57.3 (3)	C13—C14—C15—C16	−51.74 (18)
C16—C3—C4—C13	−6.0 (2)	C2—C3—C16—N1	−0.4 (2)
C2—C3—C4—C13	179.55 (18)	C4—C3—C16—N1	−175.81 (15)
C12—N2—C5—O1	169.9 (2)	C2—C3—C16—C15	−173.01 (16)
C6—N2—C5—O1	−15.1 (3)	C4—C3—C16—C15	11.5 (3)
C12—N2—C5—C4	−12.9 (2)	C1—N1—C16—C3	1.32 (19)
C6—N2—C5—C4	162.11 (16)	S1—N1—C16—C3	160.22 (13)
C3—C4—C5—O1	−35.7 (3)	C1—N1—C16—C15	173.76 (16)
C13—C4—C5—O1	−162.4 (2)	S1—N1—C16—C15	−27.3 (2)
C3—C4—C5—N2	147.29 (16)	O3—C15—C16—C3	−102.19 (19)
C13—C4—C5—N2	20.51 (19)	C14—C15—C16—C3	18.4 (2)
C12—N2—C6—C11	56.6 (3)	O3—C15—C16—N1	86.68 (19)
C5—N2—C6—C11	−117.8 (2)	C14—C15—C16—N1	−152.76 (16)
C12—N2—C6—C7	−125.4 (2)	O5—S1—C17—C18	−32.44 (18)
C5—N2—C6—C7	60.2 (3)	O6—S1—C17—C18	−166.93 (15)
C11—C6—C7—C8	0.7 (3)	N1—S1—C17—C18	82.23 (16)
N2—C6—C7—C8	−177.25 (18)	O5—S1—C17—C23	150.62 (15)
C6—C7—C8—C9	−1.7 (3)	O6—S1—C17—C23	16.13 (18)
C7—C8—C9—C10	1.1 (4)	N1—S1—C17—C23	−94.71 (16)
C8—C9—C10—C11	0.5 (4)	C23—C17—C18—C19	0.3 (3)
C7—C6—C11—C10	0.9 (4)	S1—C17—C18—C19	−176.53 (16)
N2—C6—C11—C10	178.9 (2)	C17—C18—C19—C20	0.7 (3)
C9—C10—C11—C6	−1.5 (4)	C18—C19—C20—C22	−1.8 (3)
C5—N2—C12—O2	−179.12 (19)	C18—C19—C20—C21	178.3 (2)
C6—N2—C12—O2	5.9 (3)	C19—C20—C22—C23	2.0 (3)
C5—N2—C12—C13	−0.4 (2)	C21—C20—C22—C23	−178.1 (2)
C6—N2—C12—C13	−175.46 (16)	C20—C22—C23—C17	−1.0 (3)
O2—C12—C13—C4	−168.10 (19)	C18—C17—C23—C22	−0.2 (3)
N2—C12—C13—C4	13.25 (18)	S1—C17—C23—C22	176.73 (15)

### Hydrogen-bond geometry (Å, º)

**Table:**

*D*—H···*A*	*D*—H	H···*A*	*D*···*A*	*D*—H···*A*
O4—H4*O*···O2^i^	0.82	2.00	2.7929 (19)	163

Symmetry code: (i) −*x*+2, −*y*, −*z*.
